# Bioactivity and Physicochemical Properties of Three Calcium Silicate-Based Cements: An In Vitro Study

**DOI:** 10.1155/2020/9576930

**Published:** 2020-05-22

**Authors:** Ranjdar Mahmood Talabani, Balkees Taha Garib, Reza Masaeli

**Affiliations:** ^1^Department of Conservative Dentistry, College of Dentistry, University of Sulaimani, Sulaimani-Dream Land B-25, Kurdistan Region, Iraq; ^2^Department of Oral Diagnosis, Oral and Maxillofacial Medicine in Kurdistan Board of Dental Specialties, College of Dentistry, University of Sulaimani, Kurdistan Region, Iraq; ^3^Department of Dental Biomaterials, School of Dentistry, Tehran University of Medical Sciences, Tehran, Iran

## Abstract

**Objective:**

This study evaluated the bioactivity and physicochemical properties of three commercial calcium silicate-based endodontic materials (MTA, EndoSequence Root Repair Material putty, and Biodentine™). *Material and Methods*. Horizontal sections of 3 mm thickness from 18 root canals of human teeth were subjected to biomechanical preparation with WaveOne Gold large rotary instruments. The twelve specimens were filled with three tested materials (MM-MTA, EndoSequence Root Repair Material putty, and Biodentine™) and immersed in phosphate-buffered saline for 7 and 30 days. After this period of time, each specimen of each material was processed for morphological observation, surface precipitates, and interfacial dentin using SEM. In addition, the surface morphology of the set materials, without soaking in phosphate-buffered solution after one day and after 28 days stored in phosphate-buffered saline, was evaluated using SEM; also, the pH of the soaking water and the amount of calcium ions released from the test materials were measured by using an inductively coupled plasma-optical emission spectroscopy test. Data obtained were analyzed using one-way analysis of variance and Tukey's honest significant difference test with a significance level of 5%.

**Result:**

The formation of precipitates was observed on the surfaces of all materials at 1 week and increased substantially over time. Interfacial layers in some areas of the dentin-cement interface were found from one week of immersion. All the analyzed materials showed alkaline pH and capacity to release calcium ions; however, the concentrations of released calcium ions were significantly more in Biodentine and ESRRM putty than MM-MTA (*P* < 0.05). ESRRM putty maintained a pH of around 11 after 28 days.

**Conclusion:**

Compared with MM-MTA, Biodentine and ESRRM putty showed significantly more calcium ion release. However, exposure of three tested cements to phosphate-buffered solution resulted in precipitation of apatite crystalline structures over both cement and dentin that increased over time. This suggests that the tested materials are bioactive.

## 1. Introduction

Calcium silicate-based cements used for different endodontic clinical situations such as pulp capping, apexification, root-end fillings, and perforation should possess biocompatibility, radiopacity, antibacterial activity, easy handling, good sealing ability, and bioactivity [1].

Bioactivity of a biomaterial plays a key role in tissue regeneration and healing [2]. The performance of calcium silicate materials is largely attributable to their bioactivity, that is, their capacity to release calcium ion (Ca^2+^) and produce apatite-like crystalline precipitates when in contact with phosphate-containing physiological fluids [3–8]. The precipitates are produced as a result of the dissolution of calcium hydroxide formed via hydration reactions; the resulting increase in alkalinity and Ca^2+^ concentration enhances the supersaturation of phosphate-containing fluid with respect to apatite and, hence, promotes precipitation [9].

Calcium silicate cements, such as Portland cement or mineral trioxide aggregate (MTA), currently exhibit substantial potential for use as biomaterials and have been used in a variety of endodontic applications [10, 11]. MTA is a bioactive material that features excellent apatite-forming ability [12] and exhibits excellent sealing ability, a high pH, radiopacity, biocompatibility, and an ability to stimulate dentin matrix protein expression [13–16]. However, since MTA still has some drawbacks, several new calcium silicate-based materials have been recently developed [12, 17] aimed at improving some of these drawbacks, such as discoloration potential, presence of toxic elements in the material composition, difficult handling characteristics, long setting time, high material cost, absence of a known solvent for this material, and the difficulty of its removal after curing.

MicroMega MTA (MM-MTA; MicroMega, Besançon, France), another formulation of MTA, was developed in 2011 to overcome drawbacks of the original MTA products. It is an injectable osteoconductive, osteoinductive, and biocompatible tricalcium silicate-based cement and also contains calcium carbonate, which helps in reducing the setting time [18]. The biological integration of MM-MTA is due to calcium ions, which form hydroxyapatite in contact with phosphate ions present in the body [19].

Biodentine (BD) (Septodont, Saint Maur des Fosses, France) was developed in 2009 as a novel tricalcium silicate-based cement [20]. It was described as a bioactive dentin substitute due to having similar mechanical properties to dentin. Also, it can be used with similar indications to MTA [21, 22]. Like MTA, BD shows apatite formation after immersion in phosphate solution which is indicative of its bioactivity.

An alternative material, EndoSequence Root Repair Materials putty (ESRRM putty), was developed as a premixed, injectable material formulated using bioceramic technology. It has been released by Brasseler USA (Savannah, GA) to be used as a clinical replacement for MTA. ESRRM putty has the advantages of faster setting and superior handling properties [23]. This putty possesses improved color stability and handling characteristics while exhibiting physical and chemical properties comparable with MTA. The most important property is that this material can release calcium and phosphate ions which are essential for hydroxyapatite deposition [24].

Thus, the aim of this study was to evaluate the surface texture and apatite forming ability, Ca^2+^ releasing and pH of MM-MTA, ESRRM putty, and BD in contact with phosphate-containing physiological fluids during different time periods.

## 2. Material and Method

Three calcium silicate-based materials, MM-MTA (Lot no. 71708614), EndoSequence Root Repair Material putty (Lot no. B19585), and Biodentine (Lot no. 5024200 U0), were used. The composition of the test materials is shown in Table 1. The research protocol was approved by the Ethical Committee of Sulaimani University (approval number 9, 6.2.2017). The MicroMega MM-MTA capsule (MicroMega, Besançon, France) and EndoSequence Root Repair Material putty (BC Fast Set Putty- ready-to-use; Brasseler USA-Savannah, GA) were directly used, while Biodentine (Septodont, Saint Maur de Fosses, France) was prepared according to the manufacturer's recommendations and inserted by an amalgam carrier (Shanghai, China) and condensed by a small-sized ash condenser (Shanghai, China).

### 2.1. Cement-Dentin Interface Observation

A total of eighteen caries-free single-rooted human teeth with a closed apex, extracted for different reasons, were used. The crowns of each tooth were sectioned at the cementoenamel junction using a water-cooled diamond disc at low speed. The working length for root canal instrumentation was verified by the direct method, and the length was determined to be 1 mm shorter than the actual length of the root. The root canals were instrumented by WaveOne Gold large (Dentsply Tulsa Dental, Tulsa, OK, USA) under copious irrigation with 3% sodium hypochlorite (Neo Cleaner, Neo Dental, Tokyo, Japan). The canals were then irrigated with 17% ethylenediaminetetraacetic acid (EDTA; Ultradent, South Jordan), followed by 3% sodium hypochlorite for 1 min each, washed immediately with purified water, and dried with absorbent paper points (VDW, Munich, Germany). Roots were sectioned transversely through the center of the root canal from the midroot level using a water-cooled diamond disc on a precision cut-off machine (Mecatome, Presi, France) to obtain two 3.00 ± 0.05 mm thick root sections. Root sections were randomly filled with the 3 tested materials. Any access material on the surface of all specimens was carefully removed, and the samples were wrapped in pieces of gauze soaked in phosphate-buffered saline (PBS) for 1 hr. After that, each root-filled slice was immersed individually in 2 mL of PBS solution (pH = 7.2) within sterile Eppendorf plastic tubes (Eppendorf-Elkay, Shrewsbury, MA, USA) and incubated at 37°C. The PBS was replaced every 7 days [7, 25]. After 7 and 30 days, the root sections (*n* = 3, in each period) for each material were dried at 37°C for 24 h. Specimens were then mounted on metallic stubs, gold-sputtered, and examined under a scanning electron microscope (Caesium, version 6.1.10, ID 634258760) (CS3200 Tungsten) at an accelerating voltage of 40 kV. SEM photomicrographs at several magnifications (15-3,000x) were taken to identify the formation of ultrastructure precipitates on the surface of cements and/or at the dentin-cement interfaces.

### 2.2. Surface Morphology Analyses

The topographical morphology and structural organization for the surface of the three hydrated cements were tested experimentally in six samples. Each material was placed into two plastic tubes (10 mm length, 2 mm inner diameter, and 3.8 mm outer diameter with both ends open) and allowed to set. One tube was left dry for 1 day, and the other was soaked in PBS for 28 days. At the time of examination, the wet samples were dried in a vacuum desiccator. Each tube was removed, and the drawn out cement was dissected into two planes, resulting in four pieces that represented the outer and inner surfaces on the set material. Then, they were gold-coated for SEM examination. Back-scattered images of the cement surface were taken at different magnifications under 40 kV.

The appearance of white mass or nodule on the surface of material or inside the root after exposure to PBS indicated the formation of precipitate and attributed to sealing ability of calcium silicate cements. In addition, the presence of a needle, or cubic, or a circular-spherical and lath-like crystal observed by SEM following hydration process of MM-MTA, ESRRM putty, and BD after immersion in PBS attributed to apatite forming ability and bioactivity properties [6, 12].

### 2.3. pH and Calcium Ion Release Measurement

A total of 30 plastic tubes (10 mm length, 2 mm inner diameter, both ends open) were divided into two groups: one for pH measurement and other for measuring the amount of Ca^2+^ release. Each group was further subdivided into 3 subgroups according to the material inserted (5 tubes in each). The tubes were weighed for standardization of the amount of the cement (±0.002 g) and immediately immersed in 40 mL deionized water (pH 6.9 and devoid from calcium ions), sealed with Parafilm, and kept in an incubator (37°C) throughout the study period. All laboratory equipment was previously treated with nitric acid (65%) and washed with deionized water.

The pH meter (model: PTR 79, ZAG CHEMIE Company, China) was initially standardized by buffered solutions (pH 7) and recalibrated before testing each new specimen at room temperature 25°C ± 2°C. For each sample, the pH was measured twice (calculating mean value) after specimen removal and shaking the container for 5 seconds. The pH electrode was immersed and maintained for approximately three minutes in each container while stirring to allow uniform contact with the electrode tip and avoiding air bubble formation or touching the base of the container to prevent false results. Then, the electrode was rinsed thoroughly with distilled water, dried with wipes, and reimmersed in the same sample or a new sample. The measurement was carried out at periods of 3 hours, 24 hours, 7 days, 15 days, and 30 days. The mean pH was then plotted against time.

The measurement of Ca^2+^ released in soaking water was performed after the removal of samples from the container after 1, 2, 7, and 28 days by using an inductively coupled plasma-optical emission spectroscopy test (Optima 2100 DV Perkin Elmer). HNO3 (5%) with 5, 10, and 15 ppm Ca was used as a series concentration for calibration before reading the released calcium. The amount of Ca^2+^was measured in ppm. Cumulative calcium release was calculated separately for each of the five samples of tested materials by totaling the amounts released at the four different endpoints.

### 2.4. Statistical Analysis

The normally distributed data were analyzed by a parametric test by using SPSS Statistical software version 25 for Windows (SPSS, Chicago, Il, USA). One-way analysis of variance (ANOVA) was used to conduct the statistical analysis, followed by Tukey's honest significant difference test with a significance level of 5%.

## 3. Result

### 3.1. SEM Analysis

**Figure 1:**
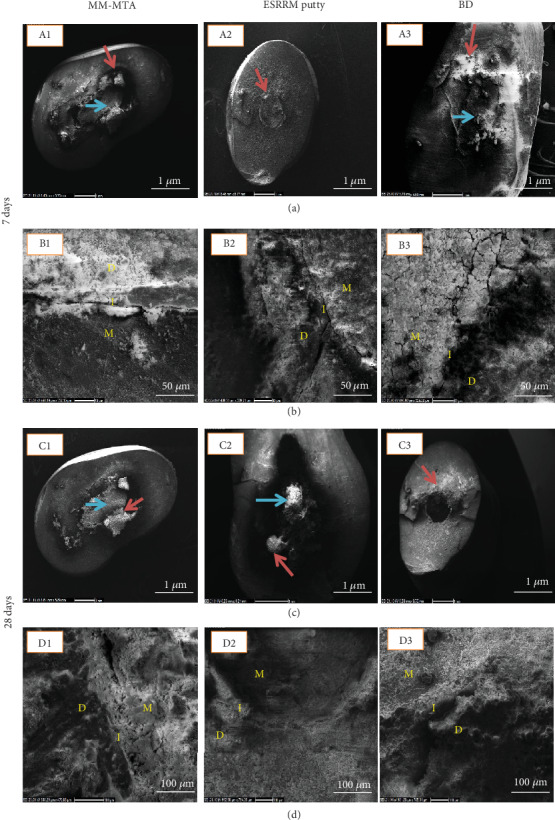
Scanning electron microscopy examination of the tested materials (1 = MM-MTA, 2 = ESRRM putty, 3 = Biodentine) immersed in phosphate-buffered saline (PBS) for 1 week ((a) low power 1 *μ*m; (b) high power 100 *μ*m) and 28 days ((c) low power 1 *μ*m; (d) high power 100 *μ*m). All specimens immersed in PBS for 1 week demonstrated formation of precipitates (blue arrow) on the material's surface. At 28 days (c), the specimens showed a large amount of precipitates (red and blue arrows). Formation of precipitates at the dentin-material interface (red arrow) extended over material and dentin surfaces (blue arrow) in some areas following one week and 28 days of immersion (c). At higher magnification, the formation of an interfacial layer could be seen at the material-dentin interface (d). M: material; I: interface; D: dentin.

Scanning electron microscopy analysis revealed an apatite crystal formation over cements along the interface (blue headed arrows) and within the interfacial dentin (red headed arrows) in all groups. There was limited precipitate formation on the surfaces of all three cements after 1 week of immersion in PBS (Figure 1(a)). After 28 days, the topography of the cements was dramatically changed, and their surfaces were covered by a substantially more considerable amount of precipitate and apatite crystal formations (Figure 1(c)). After 28 days, SEM analysis of precipitates revealed the particle morphology to be more uniform and smooth than after 1 week for MM-MTA, ESRRM putty, and Biodentine specimens.

Scanning electron microscopy evaluation of Biodentine after 1 week of immersion revealed more spherical precipitates on both the surface of the material and the dentin-material interface (Figure 1 (A3 and B3)) compared to both MM-MTA and ESRRM putty.

This interfacial zone was composed of an area devoid of larger particles but with smaller particles interspersed in the interfacial region. This was more evident in MM-MTA, ESRRM putty, and Biodentine after 28 days of immersion. The dentinal tubule penetration was less in ESRRM putty compared to the other cements after 28 days (Figure 1(d)).

**Figure 2:**
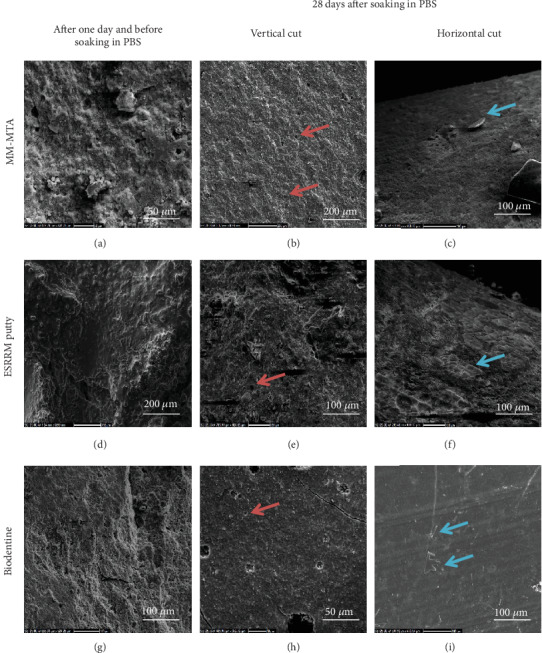
Scanning electron microscopy micrographs of set cements after one day and before soaking in PBS and after 28 days soaking in PBS at different magnification (50, 100, and 200 *μ*m).

Representative surfaces of the hydrated mixed MM-MTA, ESRRM putty, and Biodentine after one day and before soaking in PBS, as well as after storage in PBS for 28 days, are shown in (Figure 2). Hydrated ESRRM putty exhibited a small and more developed hexagonal crystal structure compared to mixed MM-MTA and Biodentine (Figures 2(d)–2(f)). However, Biodentine stored in PBS exhibited a relatively smoother surface compared to both MM-MTA and ESRRM putty (Figures 2(h) and 2(i)). A dense homogeneous structure was observed in the three set cements after 28 days in PBS, with smaller porosities in the MM-MTA and ESRRM putty compared to the Biodentine group (red headed arrows), and after 28 days of storage in PBS, the outer surface was amorphous and regular, with many visible deposits composed of aggregates of apatite nanospherulites in vertical cut geometry (Figures 2(b), 2(e), and 2(h)). Diffused larger hexagonal and cubic (needle-like) crystals (blue headed arrows) were detected on all cement surfaces after 28 days of PBS immersion in horizontal cut (Figures 2(c), 2(f), and 2(i)).

### 3.2. pH and Calcium Ion Release Measurement

**Figure 3:**
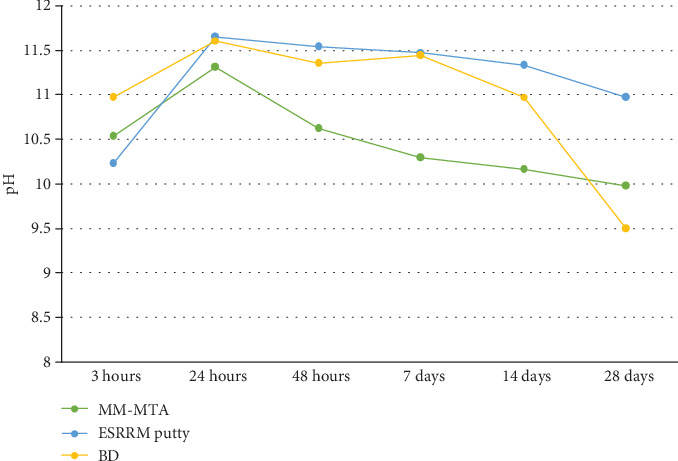
Mean pH values of soaking water of MM-MTA, ESRRM putty, and BD along different time intervals.

All materials induced alkalization of the soaking water that decreased with time but was still present at 28 days (Figure 3). They alkalized the soaking water to pH 11.7 at short times (after 24 hours), then decreasing to a pH of >9 after 28 days, with one material (ESRRM putty) maintaining a pH value of around 11.

**Figure 4:**
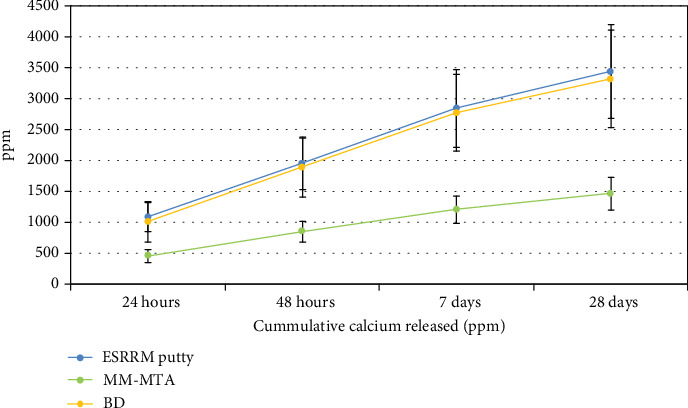
Mean of cumulative calcium released (ppm) in soaking water.

Additionally, the three tested cements released Ca^+2^ and the release decreased with the soaking time (Table 2, Figure 4). ESRRM putty and BD showed a very high Ca^+2^ release at both short (24 hours and 48 hours) and long times (28 days), while the amount of Ca leached by MM-MTA was significantly lower over time (*P* > 0.05).

## 4. Discussion

A combination of scanning electron microscopy, calcium ion release and pH analysis was used to characterize and evaluate the bioactivity and physiochemical properties of three different calcium silicate-based cements. This study clearly demonstrated that MM-MTA, EndoSequence Root Repair Material putty, and Biodentine produced apatite crystals that dramatically increased with time after immersion into a phosphate-containing solution, released Ca ions, and possessed high alkalinity, all of which indicate their bioactivity.

The superimposition of the peaks and the presence of multiple compounds within the materials of phase identification and characterization by X-ray energy dispersive analysis has been addressed by [26]. Scanning electron microscopy and an inductively coupled plasma-optical emission spectroscopy test were therefore used in the present study to allow material microstructure observation and surface visualization and to measure calcium ion release from each tested material over different time periods.

No previous study evaluated the effect of different cutting geometry (vertical and horizontal cut) of hydrated set cements before and after immersion in PBS on apatite forming ability, crystallography, and surface porosity assessment using SEM. Also, this study provides a new line for the evaluation of the surface morphology and bioactivity of three calcium silicate-based cements with different types of mixing: premixed and loaded in syringe (ready to use) (ESRRM putty), predosed and supplied in capsule (MM-MTA), and two component material powder and liquid mixing (Biodentine) in one study with two different time points (at one week and one month).

In the present study, PBS was used as a simulated tissue fluid containing phosphate for the purpose of mimicking normal in vivo conditions in laboratory studies [27]. The high amount of phosphate in PBS represents the continuous replenishment of phosphate ions from tissue fluids, and in the absence of calcium, the release of calcium ions from the cements becomes the limiting parameter in the precipitation reaction and marks the bioactivity of the experimental cement [13, 28].

In this study, a human root dentin was used to evaluate standard canal lumens containing three tested calcium silicate-based cements soaked in PBS for 7 and 28 days, a recommended method for the evaluation of hard tissue bioactivity and to obtain more clinically relevant findings [9].

All three materials produced surface precipitates and crystal apatite formation after incubation in phosphate-buffered solution as well as at the material-dentin interface for three materials; the formation of an apatite layer was detected on the surface of both Biodentine and ProRoot MTA as well as at the material-dentin interface for both materials. This is consistent with findings by a number of studies [8, 29]. Although no study has previously demonstrated the bioactivity of MM-MTA in human root dentin, Khalil et al. [30] observed the mineralogy of MM-MTA during their investigation of the physical and chemical properties of the material.

The morphological differences in the crystals may represent, at least in part, the transformation of the metastable amorphous calcium phosphate phase into an apatite phase [28]. In this study, crystalline apatite structures were observed that became larger with increased immersion time, confirming a trend noted in previous studies [31, 32]. It has been found that immediately following the hydration of tricalcium and/or dicalcium silicate, Ca and OH ions are released into the surrounding environment, resulting in the formation of a calcium hydroxide (portlandite) precipitate and calcium silicate hydrate (CSH) gel, and with increasing time, greater precipitation occurs as a result of the presence of phosphorus ions included in phosphate-containing media.

The growth of a layer of apatite on calcium silicate-based cements in phosphate-containing solutions is an ideal environment for stem cell and osteoblast differentiation and colonization to support new bone formation. Apatite together with the epigenetic signals correlated to ion release may well explain the excellent clinical outcomes [33]. Moreover, the apatite-forming ability may provide the clinical advantage of improved sealing through deposition with time of calcium phosphates at the interface and inside the dentinal tubules of the root canal [34].

Another important finding in this study related to the interfacial zone in MM-MTA, ESRRM putty, and Biodentine with the dentin interface after 28 days of immersion in PBS. This finding is also comparable to previous findings [8, 29, 35]. The interfacial precipitates have been described as hydroxyapatite- or calcium-deficient carbonated apatite (i.e. amorphous calcium phosphate (ACP)). Carbonated apatite is also known as biologic apatite and represents the mineral phase of hard tissue (bone, dentin, and cementum) [36]. The mechanisms involved in the formation of the interfacial layer include the release of Ca^2+^ ions into the phosphate-containing environment, during and after setting, and the formation of ACP precipitate which subsequently transforms into apatite crystals [3, 5]. The bioactivity of calcium silicate cement can thus be attributed to its capacity to form carbonated apatite, which is important to the formation and maintenance of the bone tissue biomaterial interface. These observations, the formation of apatite and ability to release calcium ion and high alkalinity, were confirmed on the surface of the three cements tested and can account for their potential to stimulate repair and promote hard tissue deposition, indicating their bioactivity.

The ability to release Ca^+2^ and OH− may be important if we consider the biointeractive properties of these ions. However, the increase in pH and the high concentration of Ca^2+^ ions [37] enhance the supersaturation of the solution with respect to apatite and promote the precipitation of the carbonated apatite coating layer on the cement surface.

According to the manufacturer, these cements produce high pH, nearly (12.5), but the present study found that the maximum pH value was 11.7 after 24 hours. Alkaline pH induces an antibacterial effect and favors apatite precipitation [38]. These materials were demonstrated to strongly alkalize the soaking medium in the first three hours (up to 10), reaching a maximum level after 24 hours, with values then decreasing from 48 hours. At 28 days, all the materials showed a pH > 9, with one material (ESRRM putty) maintaining a pH value of around 11. These values suggest that these materials may preserve their properties in the long term, providing support to periradicular healing processes.

All storage solutions exhibited an alkalizing pH at all time periods. BD exhibited high pH compared to MM-MTA except at the end of the interval, with MM-MTA maintaining better alkalinity than BD at 28 days, and this agrees with previous studies [30, 39].

The alkalizing activity of ESRRM putty, showing a pH > 11.7 after 1-day immersion, also supports a previous finding by Candeiro et al. [40]. Moreover, ESRRM putty showed a higher alkalizing activity in the long term: pH mean value at 28-day evaluation was 11, which is consistent with another study by Zamparini et al. [41]. On the other hand, a study done by Lee et al. [42] found a lower pH of ESRRM putty at 24 hours, with the maximum level reached at 7 days and without maintenance of a high pH at 14 days.

Calcium ion is the major element that leaches out from the set calcium silicate-based cements and interacts with the external environment to form crystalline precipitates [4, 28]. Calcium release during the hydration process is a good indicator of calcium silicate hydrates formation due to their amorphous structure [43].

In the present study, the amounts of calcium ions released from ESRRM putty and BD were significantly more than for MM-MTA over different time periods using Inductively Coupled Plasma-Atomic Emission Spectroscopy (*P* < 0.05). This result is consistent with the study by Setbon et al. [44] which observed that BD powders contained more calcium element than MM-MTA, while Abu Zeid et al. [45] showed that compared to ProRoot MTA, Biodentine specimens exhibited greater calcium release over the 28 days using the same test. On the other hand, a slightly higher amount of calcium was released from ESRRM putty than from BD over all time periods, with no statistically significant difference, and this agrees with previous studies [46, 47].

The cumulative amount of Ca^2+^ released from ESRRM putty and BD was far higher (2336.4 mg/L and 2257.29 mg/L, respectively) than that from MM-MTA (1002.42 mg/L), after 28 days. A possible explanation for the high amount of Ca^2+^ released by calcium silicate cements could be associated with the setting and hydration reactions. In 24 hours, ESRRM putty presented the highest rate of Ca^2+^ release, and this fact might be related to the time taken for the final setting of this material that occurs after nearly 200 minutes in a moist medium [48]. Moreover, the high Ca^+2^ releases in BD can be correlated with the presence of a calcium silicate component; calcium chloride fastens the hydration reaction and favors calcium phosphate deposition, while maintaining a high pH without altering the biocompatibility [49]. In addition, the presence of calcium carbonate was demonstrated to play an active role in the hydration reaction [50] by providing nucleation sites, thereby accelerating the setting kinetics [51].

Although these elements were also present in MM-MTA, the amount of calcium ions released from MM-MTA decreased over the time periods and leaching was lower compared to both ESRRM putty and BD. This may be related to the absence of dicalcium silicate in BD and ESRRM putty, which is known to be associated with a slower hydration reaction and low solubility [52].

## 5. Conclusion

Within the limitations of this in vitro study, three tested cements (MM-MTA, ESRRM putty, and BD) demonstrated the potential for bioactivity by producing apatite crystals and an interfacial layer on the root canal dentin in the simulated body fluid.

## Figures and Tables

**Table 1:** Composition of calcium silicate-based cements used in this study.

Material	Manufacturer	The composition according to manufacturer	Lot no.
MM-MTA	MicroMega, Besançon France	Powder: tricalcium silicate, dicalcium silicate, tricalcium aluminate, bismuth oxide, calcium sulfate dehydrate, and magnesium oxide. Liquid: calcium carbonate	71708614
ESRRM putty	Brasseler, Savannah, GA, USA	Tricalcium silicate, dicalcium silicate, calcium phosphate monobasic, calcium hydroxide, colloidal silica, water-free thickening agent	B19585
Biodentine	Septodont, Saint Maur des Fosses, France	Powder: tricalcium silicate (Ca_3_SiO_5_), dicalcium silicate (Ca_2_SiO_4_), calcium carbonate (CaCO_3_), iron oxide (Fe_2_O_3_), and zirconium oxide (ZrO_2_). Liquid: water (H_2_O) with calcium chloride (CaCl_2_) and soluble polymer (polycarboxylate)	5024200U0

**Table 2:** Noncumulative calcium ion release in ppm (mg/L) (mean ± standard deviation, *n* = 5 for each material) observed at the different periods (values followed by different letters in the same line (i.e., between the materials and among the times) indicate statistically significant differences according to Tukey's honest significant difference test (*P* < 0.05)).

Times	Materials			ANOVA
	MM-MTA (*n* : 5)	(ESRRMs putty) (*n* : 5)	BD (*n* : 5)	
24 hours	461.82^a^ (±104.2246)	1088.34^b^ (±238.6041)	1019.82^b^ (±325.709)	*F* = 10.188 *P* = 0.003 df = 2
48 hours	398.74^a^ (±65.08892)	872.66^b^ (±197.6176)	877.6^b^ (±159.5173)	*F* = 16.510 *P* = 0.000 df = 2
7 days	352.78^a^ (±78.03606)	887.68^b^ (±207.6355)	886.66^b^ (±151.095)	*F* = 19.823 *P* = 0.000 df = 2
28 days	260.62^a^ (±52.82828)	598.9^b^ (±127.5169)	543.76^b^ (±199.1337)	*F* = 8.417 *P* = 0.005 df = 2

## Data Availability

The data used to support the findings of this study are available from the corresponding author upon request.
